# Yttrium-90 TOF-PET-Based EUD Predicts Response Post Liver Radioembolizations Using Recommended Manufacturer FDG Reconstruction Parameters

**DOI:** 10.3389/fonc.2021.592529

**Published:** 2021-10-05

**Authors:** Michel Hesse, Philipe d’Abadie, Renaud Lhommel, Francois Jamar, Stephan Walrand

**Affiliations:** Nuclear Medicine, Cliniques universitaires Saint-Luc, Brussels, Belgium

**Keywords:** radioembolization, TOF-PET, EUD, dose-response, reconstruction

## Abstract

**Purpose:**

Explaining why ^90^Y TOF-PET based equivalent uniform dose (EUD) using recommended manufacturer FDG reconstruction parameters has been shown to predict response.

**Methods:**

The hot rods insert of a Jaszczak deluxe phantom was partially filled with a 2.65 GBq ^90^Y - 300ml DTPA water solution resulting in a 100 Gy mean absorbed dose in the 6 sectors. A two bed 20min/position acquisition was performed on a 550ps- and on a 320ps- TOF-PET/CT and reconstructed with recommended manufacturer FDG reconstruction parameters, without and with additional filtering. The whole procedure was repeated on both PET after adding 300ml of water (50Gy setup). The phantom was acquired again after decay by a factor of 10 (5Gy setup), but with 200min per bed position. For comparison, the phantom was also acquired with ^18^F activity corresponding to a clinical FDG whole body acquisition.

**Results:**

The 100Gy-setup provided a hot rod sectors image almost as good as the ^18^F phantom. However, despite acquisition time compensation, the 5Gy-setup provides much lower quality imaging. TOF-PET based sectors EUDs for the three large rod sectors agreed with the actual EUDs computed with a radiosensitivity of 0.021Gy^-1^ well in the range observed in external beam radiotherapy (EBRT), i.e. 0.01-0.04Gy^-1^. This agreement explains the reunification of the dose-response relationships of the glass and resin spheres in HCC using the TOF-PET based EUD. Additional filtering reduced the EUDs agreement quality.

**Conclusions:**

Recommended manufacturer FDG reconstruction parameters are suitable in TOF-PET post ^90^Y liver radioembolization for accurate tumour EUD computation. The present results rule out the use of low specific activity phantom studies to optimize reconstruction parameters.

## Introduction

The first dose-response dependence in liver radioembolization was obtained using ^90^Y loaded resin spheres as early as 1994 by Lau et al. in a heroic way (1): normal liver and tumour doses were measured in the catheterization room by scanning the liver surface with a calibrated intraoperative beta probe. More remarkably, small sphere amounts were sequentially injected until the planned liver dose was reached according to the beta probe measure. The 30-month patient follow-up showed a clear splitting of the survival rate for a 120Gy tumours dose threshold.

Modern imaging based dose-response correlations in ^90^Y liver radioembolization have initially been reported using tumour absorbed doses assessed with pre-therapy ^99m^Tc-MAA SPECT (2). Later, more convincing relations between tumour control probability (TCP) and absorbed dose were obtained with post-radioembolisation ^90^Y bremsstrahlung SPECT for resin spheres (3) as well as for glass spheres (4). Up to now, more dose-response correlations were reported with this last modality (5–8) than with ^99m^Tc-MAA SPECT (9, 10).

The first ^90^Y PET/CT imaging in humans in 2009 (11), triggered many phantom studies to assess the optimal PET reconstruction parameters in dose assessment post liver radioembolization (12–23). Contrasting recovery and noise level were evaluated for various sphere diameters, or vial sizes, filled with homogeneous activities modelling tumours and surrounded by a homogenous active background modelling the healthy liver parenchyma. The specific activity ratio between modelled tumours and the background ranged from 4 to 8. The modelled liver specific activity was about 1/2 to 1/8 fold that reached in typical liver radioembolization. However, due to the constant random rate generated by the natural radioactivity of lutetium based crystals (24), lower specific activity in the target of interest cannot fully be counterbalanced by increasing the acquisition time or by summing several slices. Low count rate acquisition can result in reconstruction bias (25), extra-hepatic artefactual activity (26) and reduced signal to noise ratio (SNR) (27).

Reported optimal reconstruction tradeoffs between contrast recovery and noise control ranged from 1 up to 3 iterations x 21 subsets, with or without 5 mm FWHM Gaussian post filtering (12–23). This large variation in optimal reconstruction parameters results from the different chosen contrast recovery and noise control tradeoffs, and especially from the different phantom setups and the presence or absence of TOF assessment.

Besides these phantom studies, the first reported ^90^Y TOF-PET based dose-response correlation showed that, similar to external beam radiotherapy (EBRT), the baseline haemoglobin level had a major impact on the absorbed dose efficacy (28). This impact was later confirmed in a retrospective analysis of 606 liver radioembolizations (29). Other ^90^Y PET based dose relations were reported (30–33) confirming the factor 2 ratio for efficacy and toxicity per Gy already observed in the bremsstrahlung SPECT studies between glass and resin spheres.

Recently, ^90^Y TOF-PET based equivalent uniform dose (EUD) was shown to reunify survival response observed for glass and resin spheres radioembolizations in HCC using the same 40Gy-dose threshold, similar to the one used in EBRT (34). Besides giving a better understanding of the radiobiology underlining the tumour response in radioembolization, the EUD formalism takes automatically into account the dose heterogeneity inside the tumour. Indeed, tumour doses can exhibit very different heterogeneity levels depending on the tumour vascularisation, and also on the sphere specific activity that can be finely tuned by letting the device vial decay before the catheterization. This reunification was obtained using the standard FDG reconstruction parameters, i.e. 3 iterations x 33 subsets without filtering as recommended by the considered TOF-PET manufacturer, followed by a spatial resolution recovery.

The purpose of this study was to evaluate a phantom and ascertain whether these parameters can be adapted for tumour EUD assessment in clinical statistics. To assess the reconstruction robustness versus the heterogeneity pattern, the EUD adequacy was evaluated in the six hot rod sectors of a Jaszczak deluxe phantom. The sector insert was only partially filled to get a regional count rate similar to that of radioembolized tumours.

## Material and Methods

### Phantom Setup

**Figure 1:**
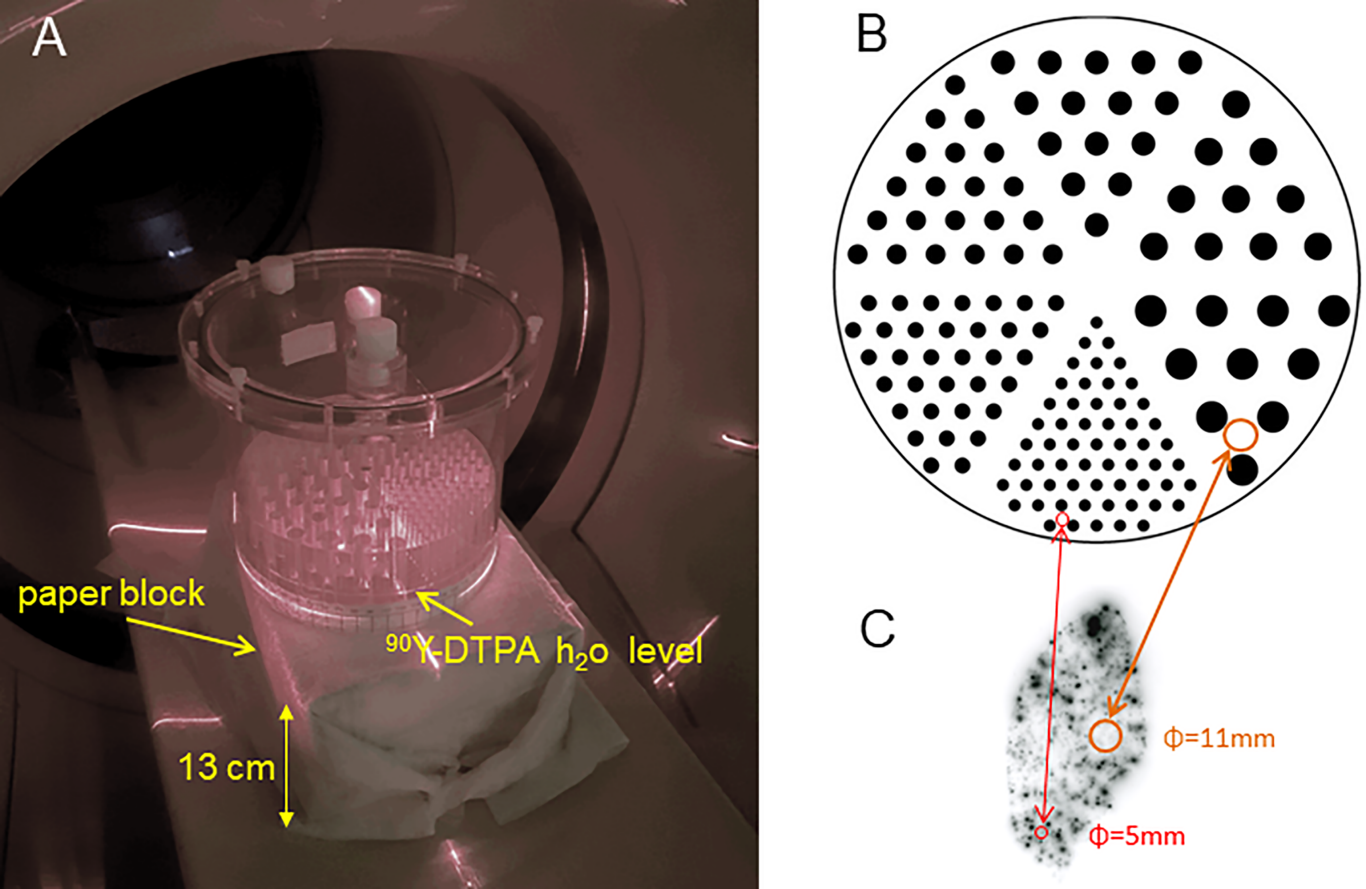
**(A)** Jaszczak deluxe phantom set in a vertical position on a paper bloc modelling the patient attenuation. Only a part of the hot rod insert was filled with an active solution to reach a typical clinical tumour absorbed dose using a 3GBq total ^90^Y activity. **(B)** Actual hot rods map. **(C)** Autoradiography of a normal liver (NL) tissue resected 9 days post ^90^Y resin spheres liver radioembolization delivering 52Gy to the NL tissue [reprinted from (35) with permission of EJNMMI]. **(B, C)** are represented at the same scale.

A Jaszczak deluxe phantom containing the hot rod insert (cylinder diameters: 4.8, 6.4, 7.9, 9.5, 11.1, 12.7 mm) was imaged set in the vertical position (**Figure 1**). The insert lay on 2mm-spacers set at the bottom of the phantom tank allowing free communication of liquid between rods. The fixation holes of the insert were filled with solid perspex cylinders to avoid extra active rods in between the six sectors.

A 2.65 GBq ^90^Y - 300ml DTPA water solution was poured into the phantom, giving an 18mm-height filling of the rods. This pouring was slowly performed using a 50 ml syringe connected to 2 catheter lines with a three-way tap, the tip of one being in the ^90^Y-DTPA container and the other one located in the 2mm-space below the sectors insert. This method prevents any air bubble formation in the rods. In a periodic hexagonal mesh, the rod volume fraction is 0.227 independently of the rod diameters. Thus, the absorbed dose D in each whole sector region, i.e. including rods and plastic, is given by the relation (6):


(1) $$D=49.67\;\times \;\frac{2.65}{0.3}\;\times 0.227\;\approx 100\;Gy$$

The phantom was set on a 13x22x29 cm^3^ paper block of 0.91g/cm^3^ density (**Figure 1A**). Taking into account the phantom top cover thickness (1 cm), the hot sector thickness above the ^90^Y solution (6 cm) and the phantom bottom wall thickness (1 cm), the total attenuation length in the vertical direction was 21cm. This attenuation is thus equivalent to that of the fully filled phantom set in the conventional horizontal position.

The acquisition-reconstruction procedure was repeated on both PETs after transferring the phantom solution into a container, pouring an additional 300ml DTPA water solution into the container, mixing the container solution, and pouring back the phantom with this new solution, resulting in a 50 Gy mean absorbed dose in the sectors. The transferring and pouring were performed using the same syringe system as for the first acquisition. The resulting fill height was 36 mm, which had a marginal impact on the phantom attenuation, as this small 18 mm increase occurs only in the exact vertical direction.

### Acquisitions

A two bed 20min/position acquisition was performed on a 550ps TOF-PET/CT [Philips Gemini TF64 (36)] and a 320ps TOF-PET/CT [Philips Vereos (37)].

The phantom was then again acquired after a 10 fold activity decay, i.e. 5 Gy mean absorbed dose in the sectors but with a 10 fold longer acquisition time, i.e. 200min per bed position.

The natural crystal radioactivity generates a constant 1400 randoms/sec in the whole field of view (FOV), i.e., after the rejection of the randoms located outside the ring diameter by the TOF information (13). Note that in TOF-PET the randoms rate contribution is reduced by the square of the ratio between the target diameter D and the FOV diameter D_FOV_, as shown by the generalized signal to noise ratio (SNR) developed by M. Conti (26) taking into account the TOF information:


(2) $$SNR^{2}\;\propto \;\frac{D}{\Delta x}\;\frac{T^{2}}{T+S+\;{\left(D/D_{FOV}\right)}^{2}\;R}$$

where *T*, *S* and *R* are true, scatter and random coincidence counts, respectively. Δx is the position uncertainty due to the TOF resolution. The randoms R can be written as:


(3) $$R=d\;2\tau \;{\left(\rho_{A}\;A\;+\;\rho_{A_{Lu}}\;A_{Lu}\right)}^{2}$$

where A is the activity to be imaged, *A_Lu_* is the ^176^Lu radioactivity present in the crystal ring, d the acquisition duration and τ the coincidence window width. The coefficients ρ take into account the emission abundance, the geometric and the crystal sensitivities. As T and S are both proportional to the activity A, eq. 2 can be rewritten as:


(4) $$SNR^{2}\;\propto \;\frac{D}{\Delta x}\;\frac{T}{1+SF/\left(1-SF\right)\;+\;{\left(D/D_{FOV}\right)}^{2}\;2\tau \;\left(\rho_{A}\;A\;+\;2\;\rho_{A_{Lu}}\;A_{Lu}+\;\rho_{A_{Lu}}^{2}\;A_{Lu}^{2}/\left(\rho_{A}A\right)\right)}$$

where *SF* is the scatter fraction, i.e., *S/(T+S).*

The last denominator term of Eq. 4 shows that SNR → 0 when A → 0, even if the acquisition duration is increased to keep T constant.

For purposes of comparison, the hot rod sectors were also acquired in a clinical ^18^FDG whole-body (WB) setup, i.e. filled with an ^18^F-FDG 5.5 MBq/l solution that corresponds to a mean liver SUV=2 in a 300MBq 75kg-patient WB study one hour post-injection. The phantom was acquired with a 1.5 min per bed position. The attenuation was similar to that of the ^90^Y phantom.

### Reconstruction

The standard FDG reconstruction parameters advised by the manufacturer were used, i.e. 4x4x4mm3 reconstruction voxel with 3 iterations x 33 subsets for the 550ps TOF-PET and 3 iterations x 15 subsets for the 320ps TOF-PET. For comparison purposes, EUDs were also computed after applying 6mm-FWHM Gaussian filtering on the reconstruction.

The 550ps TOF-PET uses a non-TOF modelled single-scatter simulation and a raw delayed windows random estimation (36). The 320ps TOF-PET uses a Monte Carlo based scatter simulation and a Casey averaged delayed windows random estimation (37). Both scatter estimations do not use sinogram tail fitting. Random estimation is introduced in the denominator of the ML step as an additional term (38). For the 550ps TOF-PET, the scatter fraction assessment according to the NU2-2001 procedure with a 20, 27 and 35 cm diameter phantoms gave an SF of about 28, 35 and 42% for the low count rate limit (36).

As the 550ps TOF-PET software does not include a resolution recovery in the reconstruction software, the Lucy-Richardson iterative deconvolution (39, 40), using a spatial invariant resolution kernel, was applied to the reconstructed slices. This spatial resolution correction was shown to provide similar recovery coefficients to those of other PET systems including a point spread function (PSF) modelling in the reconstruction (12, 13, 20). For the purpose of isolating the TOF resolution impact, the same resolution recovery was used for the 320ps TOF-PET. FDG phantoms were reconstructed in the same way.

A circular region of interest (ROI) with a diameter equal to that of the rod was drawn on each rod position. Afterwards, the mean and variance of the counts per pixel in the ROIs were computed and normalized to obtain 100 for the largest hot rod sector of each setup. This method allows it to get free of activity assessment incertitude that could impact the ^18^F-^90^Y comparison.

### Dosimetry Assessment

The voxel dose histograms were computed using a validated scheme (12, 13). The activity distribution was convolved with the ^90^Y dose kernel in the water taking into account the continuous beta energy spectrum. For the sake of presentation clarity, all reconstructed images were rescaled to the first acquisition time (decay correction lower than 3%).

Jones and Hoban (41) introduced the EUD concept which is simply the dose that is uniformly given to all cells and which should provide the same survival fraction as the set of dose *D_i_* individually given to each cell *i*. The EUD is linked to these individual doses by the relation:


(5) $$EUD=\;-\;\frac{1}{\alpha }\;ln\left(\frac{\sum_{i}^{}e^{-\alpha \;D_{i}}}{N}\right)$$

where α is the radiosensitivity of the cell line and N is the number of cells.

It is not possible to assess the individual cell doses of *D_i_* in humans and, at best, these doses can be approximated by the voxel doses. In addition, due to limited imaging spatial resolution, the number of voxels receiving the lowest doses is underestimated. Consequently, the survival fraction is also underestimated when using equation 5 with the actual cell radiosensitivity.

Chiesa et al. (4) showed that this drawback can be mitigated using an apparent radiosensitivity lower than the actual one: they found *α* ≈ 0.003 Gy^-1^ for ^99m^Tc-MAA-SPECT/CT based EUD, i.e. one order of magnitude lower than the *in vivo* cell line radiosensitivity estimated in EBRT (42): 0.01-0.04 Gy^-1^. The patient survival study (34) showed that with its better spatial resolution, the optimal radiosensitivity to reunify the response-dose between EBRT and radioembolization was 0.035 Gy-1 when using 550ps TOF-PET based EUD. This radiosensitivity, which is within the EBRT estimation range, was thus used for the TOF-PET based sector EUD assessment.

In order to evaluate the TOF-PET based EUD accuracy, the actual EUD was also computed for the six hot rod sectors. The binary sector map (1x1x1mm^3^-voxel) was rescaled to obtain the same specific activity in the rods than in the phantom. Afterwards, the resulting activity distribution was 3D convolved with the ^90^Y dose deposition kernel to get the actual dose distribution in the sectors. The EUD was computed in the ROIS dimension to ensure the sector had a ratio of 0.227 between the total rods area and the ROI area. The α value in this EUD computation was fit for matching the TOF-PET based EUDs.

## Results

**Figure 2:**
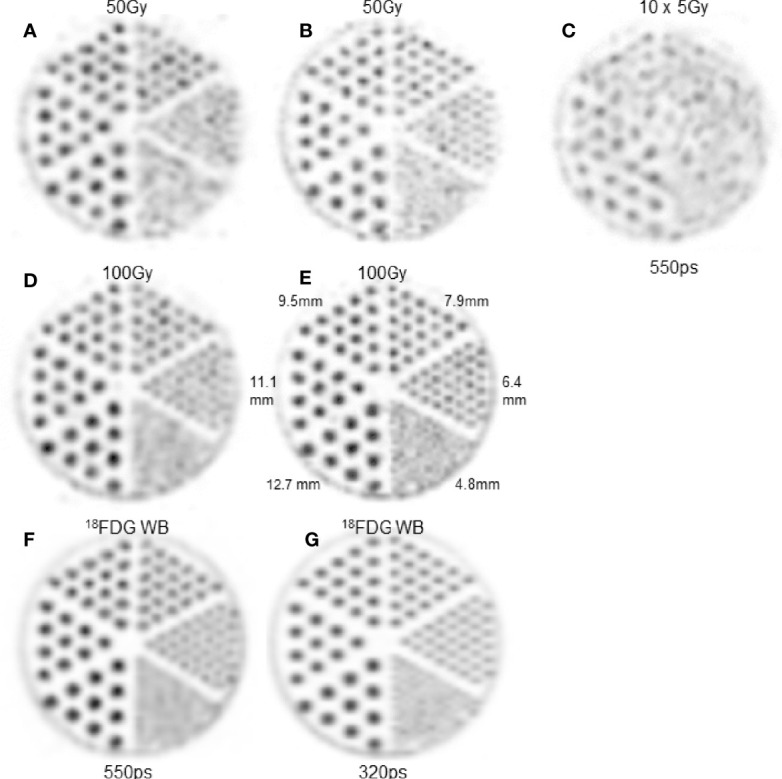
Hot rod slice. **(A, C, D, F)** 550ps TOF-PET. B,E,H: 320ps TOF-PET. **(A, B)** 4mm-thick slice in 50Gy-^90^Y-setup. **(D, E)** 4mm-thick slice in 100Gy-^90^Y-setup. **(C)** 4mm-thick slice in 5Gy-^90^Y-setup with a 10 fold longer acquisition time. Note that the acquisition time increasing **(C)** did not counterbalance the count rate reduction and result in lower image quality than **(A, D)**. **(F, G)** 4mm-thick slice in clinical ^18^FDG WB setup. As the intent is to show the capability of each modality to assess the heterogeneity pattern, the maximum voxel intensity of each image was scaled to 255.

**Figure 2** shows the hot rod slices reconstructed from the different acquisitions. The 100Gy-setup (**Figures 2D, E**) provided results almost as good as what was obtained in ^18^F phantoms (**Figures 2F, G**): rods of 4 and 5 sectors are individually visualized using the 550 and 320ps TOF-PET, respectively. Acquisition time increase in the 5Gy-setup, which was undertaken to calculate the same total number of recorded coincidences, resulted in poor quality images (**Figure 2C**).

**Table 1** clearly shows the huge dumping of the trues to randoms ratio for the 5 Gy phantom setup despite the ten-fold longer acquisition duration. This dumping factor is only 6.1 regarding that the intrinsic randoms coming from the ^90^Y activity as the square of the activity. The last column shows the relevant randoms to trues ratio impacting the phantom reconstruction according to the generalized SNR eq. 2 (D_FOV_ = 90cm for the 550ps TOF-PET).

**Table 1:** True (T) + scatter (S) coincidences numbers acquired on the 550ps TOF-PET for the three ^90^Y phantom setups.

Sector Dose[Gy]	Filled Height[mm]	Duration/Bed[min]	P[Mcts]	R[Mcts]	S[Mcts]	T[Mcts]	R/T	SNR
100	18	20	6.49	5.50	0.35	0.64	8.6	0.85
50	36	20	6.56	5.55	0.35	0.66	8.5	0.85
5	36	200	36.31	35.25	0.37	0.69	51.2	0.60

Prompts (P) and randoms (R) correspond to the counts recorded in the prompt and delayed coincidence window. S was estimated as 0.35 (P-R). (Mcts: megacounts). See the **Supplementary File** for an SNR curve estimation.

**Table 2** shows the normalized counts per pixel for the five largest hot rod sectors.

**Table 2:** Mean and variance of the counts per pixel normalized to 100 for the largest hot rod sector of each setup for the two different TOF resolution (TOFr) systems.

TOFr	Phantom	12.7 mm	11.1 mm	9.5 mm	7.9 mm	6.4 mm
550 ps	D: ^90^Y 100Gy	100.0 ± 15.9	96.2 ± 10.9	80.8 ± 11.9	68.2 ± 13.0	52.2 ± 8.9
	G: ^18^F DG WB	100.0 ± 5.5	89.0 ± 5.4	77.9 ± 6.1	63.1 ± 5.1	48.7 ± 5.3
320 ps	E: ^90^Y 100Gy	100.0 ± 7.7	109.5 ± 11.5	84.6 ± 7.7	68.7 ± 10.8	62.2 ± 11.6
	H: ^18^F DG WB	100.0 ± 7.1	89.5 ± 10.1	82.2 ± 10.5	68.0 ± 6.1	51.4 ± 5.4

**Figure 3:**
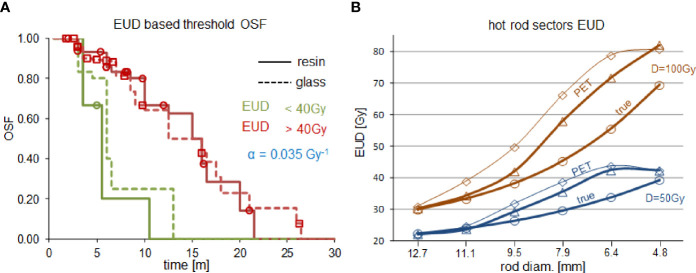
**(A)** Overall survival fraction (OSF) in HCC liver radioembolizations with resin spheres (solid line) and glass spheres (dashed line) using the same 40Gy-dose threshold on the 550ps TOF-PET based EUD:α=0.035Gy^-1^ (reprinted from (34) with permission of IOP publishing). **(B)** comparison of the 550ps TOF-PET based sectors EUD:α=0.035Gy^-1^ (triangles, diamonds are without and with 6mm-FWHM filtering, respectively) with the true sectors EUD:α=0.021Gy^-1^(circles) for a mean sector dose D of 50Gy (blue) and 100Gy (brown).

**Figure 3A** shows the dose-response reunification obtained from the two devices using 550ps TOF-PET based EUD with a common 40Gy-dose threshold in line with what is observed in EBRT (34). For rods with a diameter above 9mm, **Figure 3B** shows a good agreement between the 550ps TOF-PET based EUD (triangles) and the true sectors EUD computed with α=0.021Gy^-1^ (circles). This α value is thus the rescaling at the cell level of the apparent value 0.035Gy^-1^ observed in 550ps TOF-PET (34) and is in line with the range observed in EBRT (see **Table 1**). For smaller rod diameters a divergence was observed due to limited resolution recovery. The agreement quality is reduced by the Gaussian filtering (diamonds).

## Discussion

To the best of our knowledge, this is the first time a phantom study was conducted in order to evaluate whether valuable information from heterogeneous sources can be retrieved from ^90^Y PET imaging. We performed this analysis *via* an EUD formalism that was well adapted to predict radiobiological effects in ^90^Y radioembolization (34). The low number of injected spheres results in macroscopic heterogeneity patterns (**Figure 1C**). The impact of the heterogeneous activity distribution on the efficacy per Gy reported for high and low specific activity spheres was initially proved by Monte Carlo (43, 44) and confirmed in an animal model (45). As the dose distribution heterogeneity can vary from one patient to another, even for the same radioembolization device, it is of prime importance to evaluate whether PET imaging can assess this heterogeneity *via* the EUD formalism, explaining the choice of the hot rod phantom.

**Figure 1C** (35) clearly shows that the actual activity distribution post radioembolization is a mix of millimetric to centimetric heterogeneity pattern scales. This figure is a noise-free autoradiography showing the distribution of the actual spheres, which was further confirmed using a microscope to individually count the number of spheres number present in several activity spots (35). It is noteworthy that if the pattern scale (i.e. the inter-rods distance in the sectors, or equivalently the mean distance between sphere clusters in a tissue region) becomes lower than 4 mm, then the actual dose distribution tends to be uniform due to the ^90^Y beta range (4.3 mm mean range in water). This feature explains why the PET-based EUD overestimation is maximal (≈25%) around 6 mm (**Figure 3B**) and afterwards reduces when the activity pattern scale decreases. The higher noise present in the 50 Gy setup artificially increased the reconstructed heterogeneities and thus further decreased the EUD, which explains the concordance for the 4.8 mm rod diameter in this setup.

The present results clearly show that the EUD behaviour versus the heterogeneity scale is qualitatively reproduced using TOF-PET reconstruction with spatial resolution recovery and without any dedicated noise filtering. This observation explains why it was possible to reunify the patient survival fraction of glass and resin sphere radioembolization using TOF-PET based EUD (34). The α value used in the actual EUD computation was reduced a little bit to fit the PET based EUD. This reduction results from incomplete spatial resolution recovery joined with the reconstruction voxel size (4x4x4mm^3^). Therefore, this reduced value α=0.021Gy^-1^ can be seen as the intrinsic HCC cell radiosensitivity obtained *in vivo via* TOF-PET based EUD and is within the intrinsic range reported in EBRT [see **Table 2** in (42)], i.e. 0.01-0.04 Gy^-1^.

**Figure 3B** shows that smoothing the standard reconstruction worsens the EUD recovery using the 550ps TOF-PET. This system belongs to the first generation of TOF-PET using the raw delayed coincidence window randoms estimation. More recent systems have better TOF resolution and improved randoms estimation, such as delayed coincidence window filtering or singles based randoms estimates. Both improvements further reduce the randoms impact (eq. 2). It is thus obvious that additional noise controls, such as iteration number reduction or post-reconstruction smoothing, are also not suitable for these systems. This was also observed with the 320ps TOF-PET (see additional material). 

Recently, Dewaraja et al. (46) found that absorbed doses provided good discrimination between responding and not responding lesions. Only marginally better discrimination was obtained using the EUD formalism applied to the biological effective dose (BED). The reconstruction used 1 iteration × 21 subsets, associated with 5-mm gaussian filtering. These parameters were optimised, using clinical specific activities, for the activity recovery in uniformly active 29mL-ellipsoid and 16,8mL-spheres set in a 1200mL-liver insert of a thorax phantom, while keeping a minimal noise. The low obtained radiosensitivity value (0.0005 Gy^-1^) was similar to that obtained directly using absorbed dose, i.e., 0.001 Gy-1 (3). This value supports the conclusion that the reduced number of iterations and post-gaussian filtering does not resolve the sub-centimetric heterogeneities of the activity distribution.

The phantom reconstructions (**Figure 2**) showed that ^90^Y TOF-PET in clinical count rates provide an image qualitatively almost as good as that obtained in ^18^F phantoms. **Table 1** shows that the rod variance of the ^90^Y 100Gy setup was about 2.5 fold that of the ^18^FDG WB setup using the 550ps TOF-PET, and almost similar using the 320ps TOF-PET.

On the other hand, due to the constant random rate generated by the natural LYSO radioactivity, for a given PET-reconstruction tandem, a reduction of the specific activity cannot be counterbalanced by increasing the acquisition time (25–27), as proved by equation 4 (also see the SNR curve in the **Supplementary File**). Fortunately, toxic and efficiently absorbed doses in glass spheres radioembolization are above 100 and 200 Gy, respectively, thus in a range where the crystal radioactivity has a low impact on TOF-PET imaging.

The attenuation in the Jaszczak phantom (21 cm diameter) and NEMA 2007/IEC 2008 phantom (23×30 cm^2^) used in the QUEST study (20) are about 2.1 and 1.2 fold lower than that in a standard 70 kg male patient (20×40 cm^2^) (47), respectively. However as the present study purpose was to investigate the tumour EUD assessment accuracy, the 50 and 100 Gy sector doses were appropriate.

In future studies, targets absorbed dose in phantom modelling ^90^Y TOF-PET post liver radioembolization imaging should always be coherent with the modelling intent and be reported by the corresponding delivered dose [Gy] to the volumes of interest.

As well as the present results, showing that the ^90^Y TOF-PET post liver radioembolization does not require any special filtering in tumour EUD assessment, independent methodologies have already proved that the activity heterogeneity observed in ^90^Y TOF-PET imaging of the normal liver tissue also reflects the distribution of the actual spheres and not noise artefact. These independent methodologies were: Monte Carlo simulation of the spheres transport along the arterial hepatic tree (43, 48), autoradiography of resected liver tissue post radioembolization (35) and *in vivo* MRI imaging post ^166^Ho liver radioembolization (49).

The present study suffers from the limitation that rod sectors were used rather than grid distribution of spheres. However, this issue is mitigated for the 100Gy setup. Indeed only 18 mm rods lengths were filled, thus for the 12.7 mm diameter sector, the distribution is closer to spheres than to rods. This issue could be fully solved for any diameters in further studies using 3D printed phantoms that could better models the activity heterogeneities observed in tumour and normal liver tissue.

## Conclusions

Recommended manufacturer FDG reconstruction parameters are suitable in TOF-PET post ^90^Y liver radioembolization for accurate tumour EUD computation and normal liver tissue activity distribution assessment for the two TOF-PET’s studied. These were: 1) a first generation PMT’s system having a 550 ps TOF resolution, using a non-TOF modelled single-scatter simulation and a raw delayed window estimation (36), and 2) a last generation solid-state digital counting system with a 320 ps TOF resolution, using Monte Carlo based scatter simulation and Casey averaged delayed window estimation (37). Other reconstruction parameters and post-filtering could be adapted more to identify tumours.

This study, together with the patient survival study (34), supports the finding that EUD takes distribution heterogeneities into account due to variable microsphere decaying activities and differences in tumour vascularization.

The present phantom imaging rules out the use of low specific activity phantom studies, aiming to optimize reconstruction parameters in TOF-PET imaging post ^90^Y liver radioembolization. Increasing the acquisition time can never counterbalance the noise resulting from the constant randoms rate originating from the natural crystal radioactivity.

## Data Availability Statement

The raw data supporting the conclusions of this article will be made available by the authors, without undue reservation.

## Author Contributions

SW designed the methodology. MH prepared, acquired and processed the phantom data. Pd’A, RL, and FJ handled the medical implication of the study. All authors contributed to the article and approved the submitted version.

## Supplementary Material

The Supplementary Material for this article can be found online at: https://www.frontiersin.org/articles/10.3389/fonc.2021.592529/full#supplementary-material

## Conflict of Interest

The authors declare that the research was conducted in the absence of any commercial or financial relationships that could be construed as a potential conflict of interest.

## Publisher’s Note

All claims expressed in this article are solely those of the authors and do not necessarily represent those of their affiliated organizations, or those of the publisher, the editors and the reviewers. Any product that may be evaluated in this article, or claim that may be made by its manufacturer, is not guaranteed or endorsed by the publisher.
