# Supplementary material for: Yttrium-90 TOF-PET-Based EUD Predicts Response Post Liver Radioembolizations Using Recommended Manufacturer FDG Reconstruction Parameters
Source: Front Oncol. 2021 Oct 5;11:592529. doi: 10.3389/fonc.2021.592529 (PMC8523947; doi:10.3389/fonc.2021.592529)
Supplement: Supplementary file 1 [file DataSheet_1.docx]

**Appendix**

Figure 5: Comparison of the 32000ps TOF-PET based sectors EUD: (triangles (α=0.021Gy^-1^) and diamonds (α=0.045Gy^-1^) are without and with 6mm-FWHM filtering, respectively) with the true sectors EUD:α=0.021Gy^-1^(circles) for a mean sector dose D of 50Gy (blue) and 100Gy (brown).

Figure 5 is the equivalent of Figure 3B but for the 320ps TOF-PET system. The apparent radiosensitivity for the non-filtered and the filtered reconstruction was chosen to fit the true for the 12.7 mm rods. Similar to the 550ps TOF-PET, the post-reconstruction smoothing worsen the EUD recovery accuracy.

Figure 6: SNR estimation using Eq. 2 for the 550ps TOF-PET for different mean sector dose D aiming at an equivalent T than that of the 100 Gy phantom acquired during 20 min.

Equation 3 was rewritten as:

$R=d \left( \rho A+a_{Lu} \right)^{2}$

the parameters ρ and a_Lu_ were fitted to get the same R that those reported in Table 2.
